# Does anxiety predict the use of urgent care by people with long term conditions? A systematic review with meta-analysis

**DOI:** 10.1016/j.jpsychores.2014.06.010

**Published:** 2014-09

**Authors:** Claire Blakeley, Amy Blakemore, Cheryl Hunter, Else Guthrie, Barbara Tomenson, Chris Dickens

**Affiliations:** a Department of Psychiatry, Manchester Mental Health and Social Care Trust, UK; b NIHR School for Primary Care Research, Centre for Primary Care, Manchester Academic Health Sciences Centre, University of Manchester, Williamson Building, Oxford Road, M13 9WL, UK; c Health Services Research Unit, Nuffield Department of Population Health, University of Oxford, Rosemary Rue Building, Old Road Campus, Oxford OX3 7LF, UK; d Biostatistics Unit, Institute of Population Health, University of Manchester, Jean McFarlane Building, Oxford Road, Manchester M13 9PL, UK; e Mental Health Research Group, University of Exeter Medical School, UK; f National Institute for Health Research (NIHR), Collaboration for Leadership in Applied Health Research & Care (CLAHRC) for the South West Peninsula (PenCLAHRC), UK

**Keywords:** Anxiety, Urgent care, Long term conditions, Diabetes' asthma, Chronic obstructive pulmonary disease, Coronary heart disease

## Abstract

**Objective:**

The role of anxiety in the use of urgent care in people with long term conditions is not fully understood. A systematic review was conducted with meta-analysis to examine the relationship between anxiety and future use of urgent healthcare among individuals with one of four long term conditions: diabetes; coronary heart disease, chronic obstructive pulmonary disease and asthma.

**Methods:**

Electronic searches of MEDLINE, EMBASE, PSYCINFO, CINAHL, the British Nursing Library and the Cochrane Library were conducted These searches were supplemented by hand-searching bibliographies, citation tracing eligible studies and asking experts within the field about relevant studies. Studies were eligible for inclusion if they: a) used a standardised measure of anxiety, b) used prospective cohort design, c) included adult patients diagnosed with coronary heart disease (CHD), asthma, diabetes or chronic obstructive pulmonary disease (COPD), d) assessed urgent healthcare use prospectively. Data regarding participants, methodology, and association between anxiety and urgent care use was extracted from studies eligible for inclusion. Odds ratios were calculated for each study and pooled using random effects models.

**Results:**

8 independent studies were identified for inclusion in the meta-analysis, with a total of 28,823 individual patients. Pooled effects indicate that anxiety is not associated with an increase in the use of urgent care (OR = 1.078, p = 0.476), regardless of the type of service, or type of medical condition.

**Conclusions:**

Anxiety is not associated with increased use of urgent care. This finding is in contrast to similar studies which have investigated the role of depression as a risk factor for use of urgent care.

## Highlights

• Anxiety was not significantly associated with increased use of urgent healthcare (UC).
• Effect of anxiety was not statistically significant across different types of UC.
• Effect of anxiety was not statistically significant in different long term conditions.

## Introduction

Long term conditions (LTCs) are common and are associated with high healthcare costs. Globally, 50–80% of all healthcare spending is related to LTCs [1] with approximately 78% of the entire healthcare budget of the United States of America spent on providing healthcare for people with LTCs [2] and 69% of the healthcare budget in England allocated to the care of individuals with LTCs [3]. A disproportionate amount of healthcare costs are spent on urgent healthcare, some of which may be avoidable [4], [5], [6]. In the UK, there has been an increase in the use of urgent care over the last decade with an ever increasing number of patients presenting to Emergency Departments [7], [8], [9]. The reason for this is likely to be multi-factorial and to include factors related to organisational issues around the delivery of healthcare, disease severity, an ageing population with complex disease co-morbidity, and a variety of other, as of yet, unidentified factors.

Depression and anxiety and common co morbidities of LTCs and are associated with negative health outcomes such as: significant role impairment [10], [11], increased physical morbidity [12], [13] increased mortality [14], [15], poorer quality of life [16], [17], increased re-admission rates following hospital discharge [18], [19], [20], increased healthcare costs [21], [22], [23], and loss of work days [24], [25], [26]. However, much research has focused on the relationship between depression and LTCs, and less is known about the effect of co-morbid anxiety disorders. Anxiety disorders occur in approximately 18.1%–33% of the general population at any period of time [27], [28] and the lifetime prevalence is approximately 28.8% [27]. The prevalence of anxiety symptoms in LTCs is much higher than that in the general population, reaching up to 69% for some conditions [29], [30]. Anxiety disorders are associated with significant functional impairment and poor disease control in the context of certain LTCs [31], [32].

In a recent systematic review, it was shown that depression is associated with an increase in the use of urgent care in people with LTCs by approximately 50% [23]. However, the impact of anxiety on urgent care use remains unclear. Therefore, we have conducted a systematic review of the literature with meta-analysis to clarify the extent to which anxiety predicts urgent care use in people with LTCs.

## Method

Four non-communicable, exemplar LTCs were chosen for the purposes of the review: chronic obstructive pulmonary disease (COPD), coronary heart disease (CHD), asthma and diabetes. These four conditions contribute as the leading non-communicable causes of death worldwide, when excluding cancer [33].

The methods and results for this review are reported in line with the PRISMA Guidlelines [34].

### Eligibility criteria

The study team included papers which met the following criteria;

1. Included adults (over 18 years of age) with one or more of the following LTCs: diabetes (type 1, type 2 or unspecified), asthma (acute or chronic), COPD (acute or chronic), or CHD (myocardial infarction, stable or unstable angina), presenting results independently to any further LTMCs not included in the review criteria.
2. Prospective cohort study design.
3. Included a standardised measure of anxiety at baseline.
4. Assessed urgent healthcare use prospectively.

Urgent care was defined as any of the following: unscheduled visits to GP, consultant, or specialist nurse; visits to accident and emergency, walk in clinics, or other urgent hospitalizations; as well as costing data for these events.

In order to maximise the number of studies included within the review we did not exclude studies due to the way in which anxiety was assessed provided that patients were assessed using a valid, standardised anxiety measure. We also included all studies which used prospective, standardised measures of urgent care.

Studies were included within the review regardless of date or language of publication, sample size or length of follow up period. Papers presented in non-English languages were translated prior to screening. However, studies only available in conference abstract form, or as of the time of searching, unpublished papers, were not included in the review. This decision was made so as to ensure papers included were of high methodological quality, and typical of other published papers included. See Appendix A for full exclusion and inclusion criteria.

### Study selection

Team members with experience of conducting systematic reviews conducted in-team electronic search strategies in MEDLINE, EMBASE, PSYCHINFO, CINAHL, The British Nursing Index (using OVID search interface) and the Cochrane Library, retrieving papers from the inception of each of these databases up until the search date. Search strategies included terms of reference relevant to CHD, COPD, asthma and/or diabetes, as well as terms relevant to healthcare use, and were limited to prospective studies (see Appendix A for detailed accounts of search strategies used). As there was no medical subject heading (MeSH) terms relevant to the use of unscheduled care, electronic searches were conducted for studies relevant to all healthcare utilisation. The subset of studies that collected data on unscheduled care was identified by the research team hand-searching studies of all healthcare utilisation.

Electronic searches were first conducted in 2008 and updated periodically until 2013. Electronic searches were supplemented by hand searches of papers meeting inclusion criteria, and relevant papers were citation searched using the Social Science Citation Index.

Titles and abstracts of papers were screened by one of five researchers (AB, AK, CB, CH, and JJ) in order to identify any studies which potentially met the study inclusion criteria. Full texts of any potentially relevant papers were then screened in full by two researchers independently to assess suitability. These were then discussed in pairs, and any disagreements were resolved through discussion and/or screening by a third researcher.

**Fig. 1:**
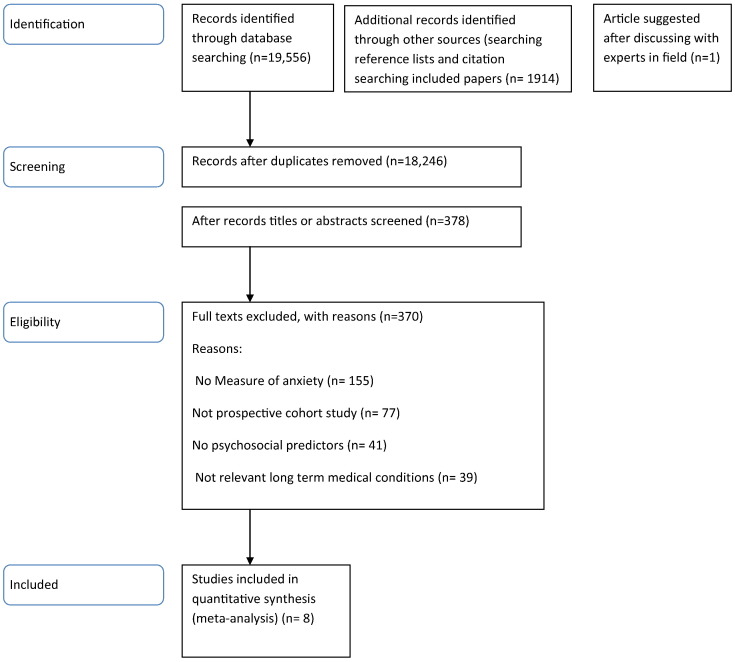
PRISMA flow diagram.

Authors were contacted for further information where results did not specify the effect of anxiety as an independent factor on the use of urgent healthcare, where healthcare was not specified as urgent or non-urgent, and where data on urgent healthcare use were not presented separately. Fifteen authors were contacted and nine responded within the pre-determined time frame of two weeks. Of the nine authors who responded, six were able to provide us with the requested data (see Appendix A for full information on studies included and excluded after author contact). Fig. 1 displays a summary of the study selection process.

### Data extraction

Standardised data extraction sheets developed by study team members and piloted on previous occasions were used to extract data from studies included in the review. Data was extracted from the studies regarding participant characteristics, measure of anxiety used, measure of urgent care use, and the strength of association between anxiety and urgent care (both univariable and multivariable associations were extracted where possible).

Primary data extraction was performed on each included paper individually by two of five researchers (AB, AK, CB, CH, JJ), and compared between two members to ensure consistency in extracted data. Any disagreements were resolved through discussion.

### Risk of bias in individual studies

The Quality Assessment Tool for Quantitative Studies was used in order to assess the methodological quality of individual studies [35], [36], dependent on whether there was:

a) An absence of selection bias for participants
b) Appropriate study design
c) Adequate control for confounding factors
d) Participants blinding to research question
e) Assessors blinding to participant's anxiety status
f) Valid and reliable data collection methods
g) Appropriate action taken for participant withdrawal
h) Appropriate use of analytical methods (The item of quality assessment relating to the integrity of interventions was excluded from the assessment).

Two researchers independently assessed the quality of studies; with consensus being reached through discussion when any disagreements occurred (the full quality assessment can be found in Table 3).

### Statistical analysis

Odds ratios (OR) and 95% confidence intervals (95% CIs) were extracted or calculated for each study where the number of participants using urgent care with and without anxiety was presented alongside the total number of subjects within each group. ORs > 1 indicated that anxiety was associated with an increased use of unscheduled care.

Where study data was presented in alternative formats (e.g. continuous data, p-value comparisons with group sizes, or as correlations), appropriate transformations were conducted using Comprehensive Meta Analysis software.

For data collected at multiple follow-up time points, ORs were calculated for the time point closest to 1 year in order to maximise consistency across studies. Where studies included more than one measure of urgent healthcare, ORs for each measure were averaged, ensuring that each study contributed only one effect measure to the meta-analysis [37]. ORs for anxiety were combined across the studies included using the DerSimonian and Laird random effects method [38], with heterogeneity among studies assessed using the Cochrane Q and I_2_ statistic [39], [40]. The I^2^ statistic is a measure of the percentage of variability in the effect estimate that is due to heterogeneity rather than due to chance. The suggested thresholds for the interpretation of I^2^ are < 25% suggesting low heterogeneity, < 50% which suggests moderate heterogeneity, and > 75% which suggests high heterogeneity [39].

Effects for anxiety are presented in both text and forest plot format.

Meta-analyses were performed using Comprehensive Meta-analysis (version 2.2.048, November 7th 2008). See Appendix A for the meta-analysis formulae used.

## Results

Eight independent studies were identified which met the criteria for inclusion [19], [20], [41], [42], [43], [44], [45], [46], providing data from a total of 28,823 participants (range n = 37 to 26,591). The eight studies were conducted in various countries; two in the United Kingdom; one in the United States of America, China, Netherlands, Germany and Canada; and one study including five Nordic countries (Finland, Norway, Iceland, Sweden and Denmark). The studies detailed patients diagnosed with COPD [19], [20], [45], [46], asthma [41], [42], [44], and CHD [43]. See Table 1 for full characteristics of the studies included.

**Table 1:** One study characteristics.

1st author and date	Condition of study	Sample size	Mean age (years)	% Males	Sample characteristics	Anxiety measure	Urgent healthcare utilisation/cost measure
[46]	COPD	26,591	69.1 (SD 11.1)	97%	Veterans with principal diagnosis of COPD exacerbation, acute or chronic bronchitis, chronic obstruction of the airway not elsewhere classified or acute and chronic respiratory failure. Excluded repeat admissions, cases with no ICD code of acute exacerbation of COPD (primary or secondary); no outpatient encounters in a year prior to admission; veterans admitted to facility with no acute care status or not initially admitted to an acute medical ward.	ICD-9	Veteran Association 30 day re-admission records
[41]	Asthma	256	56.3 (SD 16.4)	38.3%	Previously performed spirometry or broncho-provocation. Attacks of dyspnoea and wheezing or with a known allergy. Heavy smokers likely to have COPD were to be avoided.	Validated German PHQ	Patient self-reported use
[42]	Asthma	74	40.6	27%	Asthma attacks during the 20 months from October 1997. Control was selected from practice lists of patients identified as ever having asthma, only patients considered to have active asthma with duration of at least 3 years were included. The other group were patients with stable asthma matched to the other group in age, sex and BTS treatment. For more severe asthmatics, controls had to have not had an attack for a year.	7 item panic fear scale of asthma symptom checklist, practice records & ACCS	Practice records A&E attendance and hospital attendance
[43]	CHD	913	61.89 (SD 12)	64.8%	Consecutive patients who were diagnosed with MI or UA in 12 CCUs across South-central Ontario, Canada. Diagnosed with a confirmed MI or unstable angina (UA) and were 18 years of age or older. Patients who were medically unstable or unable to read or speak English were excluded.	MHQ and Anxiety Subscale of the PRIME-MD	Patient self-reports on use
[44]	Asthma	40	37.2 (SD 14)	37.5%	Diagnosis of asthma, Netherlands natives between the ages of 16 and 60 years.	ASC-PF, STAI-DY, 20 PF, and NPV	Not stated
[45]	COPD	491	Not stated	< 66%	30 years >; physician-diagnosed COPD; post bronchodilator FEV1/FVC ratio of less than 0.7 and FEV1 of less than 80% of predicted value; no fever, no worsening of respiratory symptoms, and no medication change within 4 weeks before recruitment; no primary diagnosis of asthma; no previous lung volume reduction surgery, lung transplantation, or pneumonectomy; and expected survival > 6 months.	Mandarin HADS	Patient self reports on use
[19]	COPD	416	69.2 (SD 10.5)	48.8%	Admitted for > 24 h with acute exacerbations of obstructive lung disease (asthma, chronic bronchitis, chronic obstructive bronchitis or emphysema) during the year 2000–2001. Fulfilled criteria for COPD according to the Global initiative for chronic obstructive pulmonary disease (GOLD) stage I or higher. No diagnosis of asthma.	HADS	Patient self-report on use
[20]	COPD	79	65.3 (SD 9.9)	44%	Validated diagnosis of COPD, with post-bronchodilator FEV1 < 80% of predicted, FEV/FVC ratio < 70%. MMSE > 7, Systolic BP > 100 mm Hg, white cell count (× 10^9^/1) 4–20, potassium between 3.5 and 5 mmol/l, arterial blood pH > 7.35, pO_2_ > 8 kPa, pCO_2_ < 6.7 kPa, registered with Manchester GP with adequate social support. Exclusions; suspected underlying malignancy, pneumothorax, uncontrolled atrial fibrillation, acute ECG changes, full time nursing, IV therapy, cardiac chest pain, insulin dependent diabetes, pneumonia/consolidation, chest X-ray changes, pulmonary embolism, history of falls, severe and enduring mental health problems, not English speaker.	HADS	Medical records

Anxiety was assessed using self-report questionnaires in seven out of eight studies [19], [20], [41], [42], [43], [44], [45], with one study using the ICD-9 diagnostic codes taken from patients' notes [46]. There were no cases when more than one measure of anxiety was presented. Four studies assessed the use of urgent care using self-report questionnaires [19], [41], [43], [45], and six studies used hospital admission and medical records [19], [20], [42], [43], [44], [46].

**Fig. 2:**
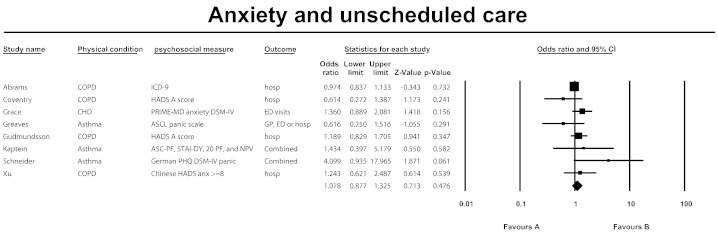
Forest plot anxiety and unscheduled care.

Of the eight studies included in the review, none showed significant effects of anxiety on the use of urgent healthcare, with only one paper showing near significant effects [41]. The independent study effects are presented in the forest plot (Fig. 2). The combined effect (OR) for anxiety across all studies included in the analysis was OR = 1.078 (95% CI 0.877–1.325), p = 0.476. Effects of individual studies showed a relatively low level of heterogeneity (Q = 9.5, d.f. = 7, p = 0.221, I^2^ = 26.07%), which is supported by the insignificance of Q. [46] included a much larger sample size than the remaining seven papers in the review, with a total of 26,591 patients, of whom 97% were male. Further sensitivity analysis was conducted in order to assess whether this study affected the meta-analysis outcome. The combined effect (OR) for anxiety across these studies was OR = 1.238 (95% CI 0.969–1.551), p = 0.087 (see Appendix A). Effects of these studies showed a very low level of heterogeneity (Q = 5.116, d.f. = 5, p = 0.402, I^2^ = 2.27%), which is again supported by the insignificance of Q.

Effects of anxiety did vary across the different types of urgent care used between studies although none of the effects were significant: ED visits [n = 1, OR = 1.360 (95% CI 0.889, 2.081), p = 0.156], hospitalisation [n = 5, OR = 1.002 (95% CI 0.876, 1.146), p = 0.976], GP visits, ED or hospitalisation [n = 1, OR = 0.616 (95% CI 0.250, 1.516), p = 0.291], and combined hospitalisation and GP visits [n = 1, OR = 4.099 (95% CI 0.935, 17.965), p = 0.061]. Comparison across groups using the analog of ANOVA revealed that these differences in effect size across various types of urgent care were not statistically significant (Q = 6.4, d.f. = 3, p = 0.093).

The effect of anxiety also varied across the different LTMCs included in the review; however none of the effects were significant: asthma [n = 3, OR = 1.355 (95% CI 0.462, 3.976), p = 0.580]; CHD [n = 1, OR = 1.360 (95% CI 0.889, 2.081), p = 0.156]; COPD [n = 4, OR = 0.998 (95% CI 0.872, 1.143) p = 0.978]. Comparison across groups revealed that these differences in effect size across the different LTCs were not statistically significant (Q = 2.1, d.f. = 2, p = 0.350).

The pooled meta-analysis was repeated for the studies grouped according to their methodological quality rating. This revealed the following effect sizes: methodologically strong studies: n = 2, OR = 0.927 (95% CI 0.701, 1.226), p = 0.594; methodologically moderate studies: n = 4, OR = 1.243 (95% CI 0.650, 2.375), p = 0.511; methodologically weak studies: n = 2, OR = 1.258 (95% CI 0.955, 1.656), p = 0.102. Comparing effects across studies grouped by methodological quality using the analog of ANOVA revealed no significant difference in the effect sizes (Q = 2.5, d.f. = 2, p = 0.288).

### Multivariable analysis

Of the 8 studies included, only 4 reported conducting multivariable analysis that controlled for severity of the LTC among other covariates {Grace et al. [43]; Abrams et al. [46]; Gudmundsson et al. [19]; Coventry et al. [20]}. These 4 studies used various measures of severity of LTC including cardiac event occurrence, length of exacerbation, St. George's Respiratory Questionnaire (SGRQ) and 30 day readmission.

Based on the reported results of multivariable analysis, where illness severity was controlled for, anxiety did contribute significantly to the multivariable models in two of the four studies. Grace et al. [43] found that depression; older age and a history of cardiovascular disease were significant predictors of self-reported recurrent cardiac events, whereas anxiety led to significantly less self-reported cardiac events (OR = 0.35, 95% CI = 0.19–0.65, p = < 0.01). Gudmundsson et al. [19] found anxiety was significantly associated with increased risk of urgent hospitalisation in a subgroup of patients who had poor health related quality of life, when analysed using Cox regression, Hazard ratio (HR) = 1.73 (95% CI, 1.18–2.53). Coventry et al. [20] found that the only significant predictors of readmission within 365 days of discharge were depression, odds ratio (OR) = 1.300 (95% CI, 1.06–1.60), p = 0.013, FEV, OR = 0.962 (95% CI, 0.93–0.99), p = 0.021, and age, OR = 1.092 (95% CI, 1.01–1.18), p = 0.026. Abrams et al. [46] found that controlling for smoking status made no changes to the effect of anxiety on risk of admission (smoking present HR = 1.22 (95% CI 1.04–1.44), smoking absent HR = 1.22 (95% CI 1.03–1.43)). See Table 2 for a full description of results.

**Table 2:** Main findings of studies included in review.

Author & date	Univariable findings	Factors controlled	Multivariable findings
[46]	Patients with anxiety were not more significantly likely to be readmitted than those without anxiety (11.3% vs. 11.5% [NS]).	Smoking status.	No significant difference in risk of admission regardless of smoking status. Smoking present HR = 1.22, 95% CI 1.04–1.44, smoking absent HR = 1.22, 95% CI 1.03–1.43.
[41]	Panic disorder did not predict hospitalisation (OR = 3.5, 95% CI = 0.7–18.3, p = 0.145), but did predict emergency visits (OR = 4.8, 95% CI 1.3–17.7, p = 0.019).		
[42]	There was no main effect of panic (p > 0.05).		
[43]	Anxious patients (1.11 [1.57]) reported more visits to the emergency department than non anxious (0.83 [1.18]) patients (t = − 1.37, p = 0.17). However, this was NS.	Age, family history of CVD, depression, Killip class, sex, family income, smoking status, diabetes and phobic anxiety.	Age (OR = 1.02, 95% CI 1.00–1.05, p = 0.05), family history of CVD (OR = 1.63, 95% CI 1.04–2.54, p = 0.03), depression (OR = 1.07, 95% CI 1.03–1.12, p = < 0.01) and prime-MD anxiety at 6 months (OR = 0.35, 95% CI = 0.19–0.65, p = < 0.01), were all significant predictors of self-reported recurrent cardiac events. All other factors NS.
[44]	State and trait anxiety not associated with increased length of hospitalisations. State anxiety not significantly associated with readmission, however trait anxiety had slight effect (1-tailed t = 1.72, p = 0.048).		
[45]	Anxiety not associated with increased risk of urgent hospital admission (p = 0.11), however length of exacerbation in days was longer for patients with anxiety than for those without (p = 0.03).	Age, sex, smoking, marital status, education, employment, living situation, FEV1, dyspnoea score, six-minute-walk distance, social support, chronic obstructive pulmonary disease-specific self-efficacy, significant comorbidities, hospital type, use of long-acting bronchodilator and inhaled corticosteroid, long-term oxygen therapy and past hospitalisation.	Anxiety was not associated with hospitalisation: Incidence Rate Ratio = 1.63 (0.88 to 3.03) for HADS anxiety ≥ 11, or for lengthy of hospitalisation for those readmitted: IRR = 1.99 (0.59 to 6.72).
[19]	Anxiety had no significant effect on rehospitalisation (p = 0.61). No significant difference between HADS anxiety scores for those who were readmitted (7.1 [4.3]) and those who were not (6.7 [4.0], p = 0.28)	Age smoking status, FEV, SGRQ.	Significant association between the HAD anxiety score and the risk of re-admission in patients with a low health status (HR = 0.81 95% CI = 0.63–1.04). In the same group, anxiety (HADS score ≥ 8) was related to increased risk of rehospitalisation (HR = 0.43 95% CI = 0.25–0.74).
[20]	No significant difference between HADS anxiety scores for those who were readmitted (8.53 ± 4.2) and those who were not (9.47 ± 4.6, p = 0.407)	Age, race, gender, individual medical comorbidities and laboratory values.	Depression (OR = 1.300, (95% CI, 1.06–1.60), p = 0.013), FEV score (OR = 0.962, (95% CI, 0.93–0.99), p = 0.021), and age (OR = 1.092, (95% CI, 1.01–1.18), p = 0.026) were the only significant predictors of readmission. Anxiety was insignificant.

### Risk of bias within individual studies

Details of the quality of studies included within the analysis are presented in Table 3. Two studies were rated as strong (no weak ratings) [20], [46], four were rated as moderate (one weak rating) [41], [42], [44], [45] and two were rated as weak (more than one weak rating) [19], [43].

**Table 3:** Quality assessment.

Author & date	Selection bias	Design	Confounding	Blinding	Data collection	Drop outs	Global rating	Discrepancy between reviewers	Reasons for discrepancy	Final rating
[46]	1	2	1	2	1	1	1	N		1
[41]	3	2	2	2	1	2	2	N		2
[42]	2	2	3	2	1	1	2	Y	Blinding procedure	2
[43]	3	2	1	2	3	2	3	N		3
[44]	1	2	3	2	2	1	2	N		2
[45]	3	2	1	2	1	1	2	N		2
[19]	3	2	1	2	1	3	3	N		3
[20]	2	2	2	2	1	2	1	N		1

### Publication bias

**Fig. 3:**
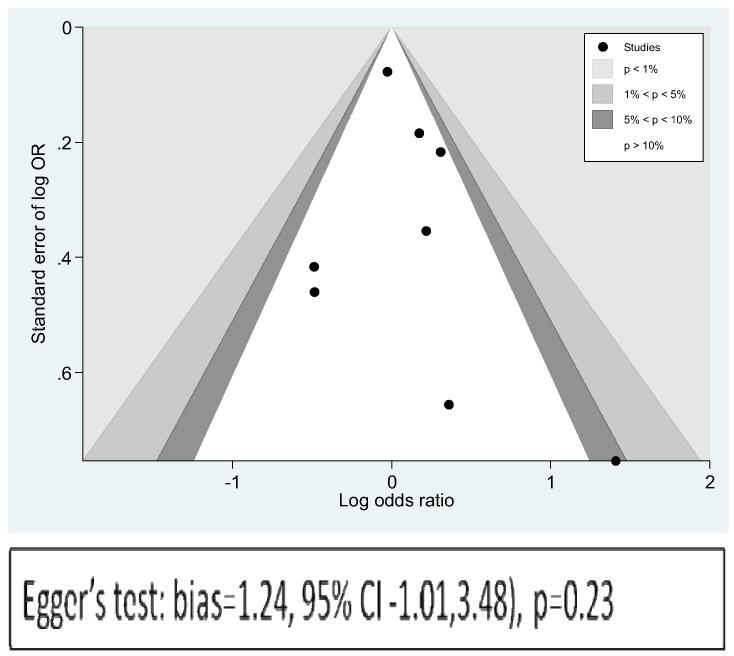
Contour enhanced funnel plot: log_e_OR vs standard error of log_e_OR.

The contour enhanced funnel plot did not appear to be asymmetrical, except for one small negative study (Fig. 3), and Egger's regression method confirmed the lack of association between log_e_OR and standard error of log_e_OR. Egger's bias = 1.24, 95% CI − 1.01 to 3.48, p = 0.23.

The Duval and Tweedie trim and fill procedure created just 1 imputed study, giving a revised random effects combined odds ratio for anxiety across all studies of 1.05 (95% CI 0.82 to 1.33), p = 0.69 (see Appendix A). This is only very slightly reduced compared with the original, and still not significant. The heterogeneity between studies is increased slightly, and still significant (Q = 12.9, df = 8, p = .040).

## Discussion

A systematic review with meta-analysis was conducted to determine whether anxiety is associated with the use of urgent care in patients with LTCs. On combining univariate findings from 8 independent studies, anxiety was not significantly associated with increased use of urgent healthcare. Effects for anxiety were also not statistically significant across different types of urgent healthcare use or different LTCs. Our findings were not significantly influenced by studies with low methodological quality. Three out of the four studies presenting multivariable analyses suggested anxiety had some effect on either urgent healthcare use, exacerbation in days, or recurrent illness, independent of severity of anxiety. However, these three studies which reported positive associations had relatively poorer methodological quality than the study which reported no such findings [20].

Our review has several strengths. First we conducted extensive searching of key electronic databases and sought expert advice from professionals within the area on potentially relevant studies. This enabled us to identify as many relevant studies as possible. Furthermore, to increase the identification of relevant papers, we chose not to limit search terms to urgent healthcare, instead we kept search terms broad, searching all healthcare use first and then hand-searching the results to find papers relevant to urgent care. Our methodological quality was further enhanced by inclusion of all relevant papers, regardless of year of publication, language of publication, sample size, or duration of follow-up. Data extraction was conducted by independent researchers with findings compared between researchers, to ensure reliability of results. We believe that our methodological rigour in identifying relevant papers contributed to the homogeneity of our findings within the analysis.

Our review has several limitations. Firstly, our decision to limit the review to four exemplar LTCs means that our findings may not be generalisable to all LTC populations. However, COPD, CHD, asthma and diabetes are all common conditions [33] with relatively high levels of psychological morbidity [47]. Three of the four LTCs are considered to be among the most burdensome non-communicable diseases worldwide [33] and are among the leading patient discharge diagnoses from emergency departments [48].

A second limitation is that we rated studies using a quality scoring system, which categorised studies according to the number of ‘weak’ characteristics displayed within the paper. The main advantage of doing this is that it allowed us to conduct sensitivity analyses to investigate the impact of quality on the observed effects of anxiety on urgent healthcare use. We recognise that in presenting quality extraction data in this way, it could be argued that we assumed all methodological weaknesses to carry an equal weight, so we have also included within the paper a table displaying how each study was scored, to enable readers to interpret quality themselves. Finally, the number of relevant papers included in this paper was quite small, with the result that our meta-analysis lacked statistical power. However, we do not think that a lack of power has affected the main result; OR for anxiety on urgent healthcare = 1.08 is very small and of little if any clinical significance irrespective of the statistical significance. Some of the subgroup analyses, for example, those demonstrating differences in effect across type of urgent care or type of long term condition, were quite large in magnitude (differences in odds ratios up to 70%) but failed to reach statistical significance, which may, at least in part, be due to a lack of statistical power.

Our original hypothesis was informed by literature which did not meet the criteria for inclusion in this review but suggested that anxiety significantly increased the healthcare use of some patients with our four specified LTCs [49], [50], [51], [52], as well as in other LTCs such as irritable bowel syndrome (IBS) [53] and sickle cell disease [54]. Whilst differences between different LTCs may be expected, the findings suggestive of a link between anxiety and urgent care in our 4 exemplar conditions, primarily arise from studies that have employed a cross sectional design. Our findings from this systematic review, suggest that the relationship between anxiety and use of urgent care becomes much weaker, if patients are studied prospectively. This is supported by findings from prospective cohort studies which have investigated other LTCs [55], [56] which suggest anxiety does not play a role in influencing urgent healthcare use.

Anxiety is clearly associated with a variety of poor outcomes in people with LTCs [31], [32], [65], [66]. A possible explanation for the ‘lack of effect’ of anxiety on urgent care may be that it is difficult to disentangle the impact of anxiety on healthcare use from the effects of co-morbid depressive symptoms. In a previous systematic review and meta-analysis depression was shown to be associated with an increased risk of up to 50% in the use of urgent care in patients with LTCs [23]. Depression and anxiety are highly correlated [57], [58] and often co-occur [59]. However, our findings suggest that there may be characteristics specific to depression that lead to a greater use of urgent care; characteristics which are not found in anxiety. It is possible that depression results in greater self neglect [60], [61] and less adherence with routine treatment [62], [63], which then leads on to more acute illness exacerbations and greater need for urgent care. Depression is also associated with negative self efficacy, which may make it particularly difficult for people to cope at times of ‘health crises’ [64].

Anxiety may only present a significant effect on use of urgent care within people with LTCs when associated with other psychological factors such as health related quality of life [67], [68]. Or it may be that certain anxiety disorders, such as panic disorder, have a greater impact on use of urgent care than generalised anxiety [69]. It was not possible to address these questions within the scope of this review due to the relatively small number of studies we identified. However, as the literature develops in this area, future reviews could use techniques such as meta-regression to tease out the impact of a wider range of more specific psychological variables on use of urgent care.

Although our findings suggest anxiety is not associated with use of urgent care, it is associated with many other adverse outcomes. There remains a requirement to identify and treat anxiety in people with LTCs.
