# Supplementary material for: Does anxiety predict the use of urgent care by people with long term conditions? A systematic review with meta-analysis
Source: J Psychosom Res. 2014 Sep;77(3):232–9. doi: 10.1016/j.jpsychores.2014.06.010 (PMC4153376; doi:10.1016/j.jpsychores.2014.06.010)
Supplement: Supplementary file 1 — Supplementary material. [file mmc1.doc]

**Appendix**

Table of contents

[1. PICO criteria for systematic review 2](#__RefHeading___Toc390789260)

[2.Electronic Search Strategies 3](#__RefHeading___Toc390789261)

[3.Additional information provided by authors 15](#__RefHeading___Toc390789262)

[4 Forrest Plot Anxiety and unscheduled care, without Abrams 16](#__RefHeading___Toc390789263)

[5 Funnel Plot of Precision by Log odds ration 16](#__RefHeading___Toc390789264)

[6 Duvall & Tweedie Trim & Fill Procedure Funnel Plot 17](#__RefHeading___Toc390789265)

[7 Formulae used in meta-analysis 18](#__RefHeading___Toc390789266)

# PICO criteria for systematic review

**Study type:** Prospective cohort study

**Population:** Adults from the age of 18, no upper age limit. Could be recruited from home, primary care, secondary care, emergency care. Must have one or more of the following long term conditions:

Diabetes: type 1, type 2 or unspecified

Asthma: acute or chronic

COPD: chronic, acute exacerbations

CHD: could be acute coronary syndrome (any of MI, stable or unstable angina), patients recruited with a diagnosis of heart failure of those recruited from cardiac rehabilitation could be included if 80% or more have diagnosis of CHD.

If the study population includes several chronic conditions, at least one of the conditions must be one of the four above, and data for one of the 4 conditions above must be presented independent of any conditions not in the list above.

**Predictors:** Anxiety; measures must be standardised.

**Outcomes:** For inclusion studies must have at least one of:

1. Prospective measures of urgent health care utilization e.g. number of unscheduled visits to GP, consultant, specialist nurse, Emergency Room visits, walk in clinic attendance, urgent hospitalizations,
2. Prospective measures of urgent health care costs

For all urgent healthcare contacts there must be an indication (in the paper or from the author by personal communication) these episodes of healthcare were urgent / unscheduled or at least precipitated by an acute episode of illness.

Unpublished studies and those published in abstract form only were not included in this review.

# 2.Electronic Search Strategies

**MEDLINE Search strategy**

Levels A (health care ut) AND C (chronic diseases) AND D (Longitudinal or Prospective or RCT) using ADJn operator AND HEALTH CARE COSTS AND EXTRA SEARCH TERMS.

| 1. Health Facilities/ut [Utilization] |
| --- |
| 2. Health Care Costs/ |
| 3. emergency service, hospital/ut [Utilization] |
| 4. outpatient clinics, hospital/ut [Utilization] |
| 5. psychiatric department, hospital/ut [Utilization] |
| 6. Hospitals/ut [Utilization] |
| 7. Health Services/ut [Utilization] |
| 8. community health services/ut [Utilization] |
| 9. community mental health services/ut [Utilization] |
| 10. Emergency Medical Services/ut [Utilization] |
| 11. emergency service, hospital/ut [Utilization] |
| 12. Triage/ut [Utilization] |
| 13. after-hours care/ut [Utilization] |
| 14. "delivery of health care_ integrated"/ut [Utilization] |
| 15. patient care team/ut [Utilization] |
| 16. primary health care/ut [Utilization] |
| 17. (unscheduled adj5 care).mp. [mp=title, original title, abstract, name of substance word, subject heading word] |
| 18. (walk-in adj5 centre).mp. |
| 19. (walk in adj5 centre).mp. |
| 20. (walk-in adj5 centres).mp. |
| 21. (walk in adj5 centres).mp. |
| 22. (walk-in adj5 clinic).mp. |
| 23. (walk in adj5 clinic).mp. |
| 24. (walk-in adj5 clinics).mp. |
| 25. (walk in adj5 clinics).mp. |
| 26. (drop-in adj5 centre).mp. |
| 27. (drop in adj5 centre).mp. |
| 28. (drop-in adj5 centres).mp. |
| 29. (drop in adj5 centres).mp. |
| 30. (drop-in adj5 clinic).mp. |
| 31. (drop in adj5 clinic).mp. |
| 32. (drop-in adj5 clinics).mp. |
| 33. (drop in adj5 clinics).mp. |
| 34. (out-of-hours adj5 service).mp. |
| 35. (out-of-hours adj5 services).mp. |
| 36. ((out of hours adj5 service) or (out of hours adj5 services)).mp. |
| 37. Hospitalization/ut [Utilization] |
| 38. hospitalization.mp. |
| 39. hospitalisation.mp. |
| 40. (emergency adj5 department).mp. [mp=title, original title, abstract, name of substance word, subject heading word] |
| 41. (emergency adj5 departments).mp. [mp=title, original title, abstract, name of substance word, subject heading word] |
| 42. (accident and emergency).mp. [mp=title, original title, abstract, name of substance word, subject heading word] |
| 43. (healthcare adj5 utilisation).mp. [mp=title, original title, abstract, name of substance word, subject heading word] |
| 44. (healthcare adj5 utilization).mp. [mp=title, original title, abstract, name of substance word, subject heading word] |
| 45. (health care adj5 utilisation).mp. |
| 46. (health care adj5 utilization).mp. [mp=title, original title, abstract, name of substance word, subject heading word] |
| 47. 1 or 2 or 3 or 4 or 5 or 6 or 7 or 8 or 9 or 10 or 11 or 12 or 13 or 14 or 15 or 16 or 17 or 18 or 19 or 20 or 21 or 22 or 23 or 24 or 25 or 26 or 27 or 28 or 29 or 30 or 31 or 32 or 33 or 34 or 35 or 36 or 37 or 38 or 39 or 40 or 41 or 42 or 43 or 44 or 45 or 46 |
| 48. Asthma/ or asthma.mp. |
| 49. Pulmonary Disease, Chronic Obstructive/ or chronic obstructive pulmonary disease.mp. or COPD.mp. or (COAD adj5 airways).mp. |
| 50. Cardiovascular Diseases/ or cardiovascular diseases.mp. or cardiovascular disease.mp. |
| 51. Diabetes Mellitus, Type 1/ or Diabetes.mp. or Diabetes Mellitus, Type 2/ or Diabetes Mellitus/ |
| 52. Diabetes Complications/ |
| 53. (Long-term conditions or long term conditions).mp. |
| 54. (Long-term health problems or long term health problems).mp. |
| 55. Chronic Disease/ or chronic disease.mp. or chronic diseases.mp. |
| 56. (Chronic illness or chronic illnesses).mp. |
| 57. (Chronic disease adj5 management).mp. |
| 58. 48 or 49 or 50 or 51 or 52 or 53 or 54 or 55 or 56 or 57 |
| 59. longitudinal studies/ or prospective studies/ or randomized controlled trials as topic/ or randomised controlled trials as topic/ |
| 60. 47 and 58 and 59 |

**CINAHL Search Strategy**

Using ADJn operator

| 1. exp Health Resource Utilization/ |
| --- |
| 2. exp Health Care Costs/ |
| 3. exp HOSPITALIZATION/ |
| 4. 1 or 2 or 3 |
| 5. (healthcare adj5 utilization).mp. [mp=title, original title, abstract, name of substance word, subject heading word] |
| 6. (healthcare adj5 utilisation).mp. [mp=title, original title, abstract, name of substance word, subject heading word] |
| 7. (healthcare adj5 costs).mp. [mp=title, original title, abstract, name of substance word, subject heading word] |
| 8. (health care adj5 utilization).mp. [mp=title, original title, abstract, name of substance word, subject heading word] |
| 9. (health care adj5 utilisation).mp. [mp=title, original title, abstract, name of substance word, subject heading word] |
| 10. (health care adj5 costs).mp. [mp=title, original title, abstract, name of substance word, subject heading word] |
| 11. hospitalization.mp. |
| 12. hospitalisation.mp. |
| 13. (hospital adj5 utilization).mp. [mp=title, original title, abstract, name of substance word, subject heading word] |
| 14. (hospital adj5 utilisation).mp. |
| 15. (outpatient department adj5 utilization).mp. [mp=title, original title, abstract, name of substance word, subject heading word] |
| 16. (outpatient department adj5 utilisation).mp. |
| 17. (emergency care adj5 utilization).mp. [mp=title, original title, abstract, name of substance word, subject heading word] |
| 18. (emergency care adj5 utilisation).mp. |
| 19. (emergency service adj5 utilization).mp. [mp=title, original title, abstract, name of substance word, subject heading word] |
| 20. (emergency service adj5 utilisation).mp. |
| 21. (emergency medical services adj5 utilization).mp. [mp=title, original title, abstract, name of substance word, subject heading word] |
| 22. (emergency medical services adj5 utilisation).mp. |
| 23. ((accident and emergency) adj5 utilization).mp. [mp=title, original title, abstract, name of substance word, subject heading word] |
| 24. ((accident and emergency) adj5 utilisation).mp. |
| 25. (unscheduled care adj5 utilization).mp. [mp=title, original title, abstract, name of substance word, subject heading word] |
| 26. (unscheduled care adj5 utilisation).mp. |
| 27. (unscheduled care adj5 costs).mp. [mp=title, original title, abstract, name of substance word, subject heading word] |
| 28. (community care adj5 utilization).mp. [mp=title, original title, abstract, name of substance word, subject heading word] |
| 29. (community care adj5 utilisation).mp. |
| 30. (community health services adj5 utilization).mp. [mp=title, original title, abstract, name of substance word, subject heading word] |
| 31. (community health services adj5 utilisation).mp. |
| 32. (primary health care adj5 utilization).mp. [mp=title, original title, abstract, name of substance word, subject heading word] |
| 33. (primary health care adj5 utilisation).mp. |
| 34. (mental health services adj5 utilization).mp. [mp=title, original title, abstract, name of substance word, subject heading word] |
| 35. (mental health services adj5 utilisation).mp. |
| 36. ((walk-in adj5 centre) or (walk in adj5 centre) or (walk-in adj5 centres) or (walk in adj5 centres)).mp. |
| 37. ((walk-in adj5 clinic) or (walk in adj5 clinic) or (walk-in adj5 clinics) or (walk in adj5 clinics)).mp. |
| 38. ((drop-in adj5 clinic) or (drop in adj5 clinic) or (drop-in adj5 clinics) or (drop in adj5 clinics)).mp. [mp=title, original title, abstract, name of substance word, subject heading word] |
| 39. ((out-of-hours adj5 service) or (out-of-hours adj5 services)).mp. |
| 40. ((out of hours adj5 service) or (out of hours adj5 services)).mp. |
| 41. 5 or 6 or 7 or 8 or 9 or 10 or 11 or 12 or 13 or 14 or 15 or 16 or 17 or 18 or 19 or 20 or 21 or 22 or 23 or 24 or 25 or 26 or 27 or 28 or 29 or 30 or 31 or 32 or 33 or 34 or 35 or 36 or 37 or 38 or 39 or 40 |
| 42. 4 or 41 |
| 43. exp ASTHMA/ or asthma.mp. |
| 44. (COPD or chronic obstructive pulmonary disease).mp. or exp Lung Diseases, Obstructive/ or COPD.mp. or (COAD adj5 airways).mp. |
| 45. cardiovascular disease.mp. or exp Cardiovascular Diseases/ |
| 46. diabetes mellitus/ or diabetes mellitus, insulin-dependent/ or diabetes mellitus, non-insulin-dependent/ or diabetic angiopathies/ or diabetic coma/ or diabetic ketoacidosis/ or diabetic nephropathies/ or diabetic neuropathies/ |
| 47. (Long-term conditions or long term conditions).mp. |
| 48. (Long-term health problems or long term health problems).mp. |
| 49. exp chronic disease/ or chronic disease.mp. |
| 50. (Chronic illness or chronic illnesses).mp. |
| 51. (Chronic disease adj5 management).mp. |
| 52. 43 or 44 or 45 or 46 or 47 or 48 or 49 or 50 or 51 |
| 53. longitudinal studies.mp. or exp Prospective Studies/ |
| 54. exp Clinical Trials/ or randomised controlled trials.mp. or randomized controlled trials.mp. |
| 55. 53 or 54 |
| 56. 42 and 52 and 55 |

**Embase search strategy**

Using ADJn operator AND extra search terms.

| 1. exp Health Care Utilization/ |
| --- |
| 2. exp "Health Care Cost"/ |
| 3. exp Hospital Utilization/ |
| 4. 1 or 2 or 3 |
| 5. (health care adj5 utilisation).mp. |
| 6. (health care adj5 utilization).mp. |
| 7. (healthcare adj5 utilisation).mp. [mp=title, original title, abstract, name of substance word, subject heading word] |
| 8. (healthcare adj5 utilization).mp. [mp=title, original title, abstract, name of substance word, subject heading word] |
| 9. (hospital adj5 utilisation).mp. |
| 10. (hospital adj5 utilization).mp. [mp=title, original title, abstract, name of substance word, subject heading word] |
| 11. (outpatient department adj5 utilisation).mp. |
| 12. (outpatient department adj5 utilization).mp. [mp=title, original title, abstract, name of substance word, subject heading word] |
| 13. (emergency health services adj utilisation).mp. |
| 14. (emergency health services adj utilization).mp. [mp=title, original title, abstract, name of substance word, subject heading word] |
| 15. (emergency ward adj5 utilisation).mp. |
| 16. (emergency ward adj5 utilization).mp. [mp=title, original title, abstract, name of substance word, subject heading word] |
| 17. (emergency care adj5 utilisation).mp. |
| 18. (emergency care adj5 utilization).mp. [mp=title, original title, abstract, name of substance word, subject heading word] |
| 19. (emergency service adj5 utilisation).mp. |
| 20. (emergency service adj5 utilization).mp. [mp=title, original title, abstract, name of substance word, subject heading word] |
| 21. (emergency medical services adj5 utilisation).mp. |
| 22. (emergency medical services adj5 utilization).mp. [mp=title, original title, abstract, name of substance word, subject heading word] |
| 23. ((accident and emergency) adj5 utilisation).mp. [mp=title, original title, abstract, name of substance word, subject heading word] |
| 24. ((accident and emergency) adj5 utilization).mp. [mp=title, original title, abstract, name of substance word, subject heading word] |
| 25. (unscheduled care adj5 utilisation).mp. |
| 26. (unscheduled care adj5 utilization).mp. [mp=title, original title, abstract, name of substance word, subject heading word] |
| 27. (unscheduled care adj5 costs).mp. [mp=title, original title, abstract, name of substance word, subject heading word] |
| 28. (unscheduled adj5 care).mp. [mp=title, original title, abstract, name of substance word, subject heading word] |
| 29. (community care adj5 utilisation).mp. |
| 30. (community care adj5 utilization).mp. [mp=title, original title, abstract, name of substance word, subject heading word] |
| 31. (community health services adj5 utilisation).mp. |
| 32. (community health services adj5 utilization).mp. [mp=title, original title, abstract, name of substance word, subject heading word] |
| 33. (primary health care adj5 utilisation).mp. |
| 34. (primary health care adj5 utilization).mp. [mp=title, original title, abstract, name of substance word, subject heading word] |
| 35. (mental health services adj5 utilisation).mp. |
| 36. (mental health services adj5 utilization).mp. [mp=title, original title, abstract, name of substance word, subject heading word] |
| 37. ((walk-in adj5 centre) or (walk in adj5 centre) or (walk-in adj5 centres) or (walk in adj5 centres)).mp. |
| 38. ((walk-in adj5 clinic) or (walk in adj5 clinic) or (walk-in adj5 clinics) or (walk in adj5 clinics)).mp. |
| 39. ((drop-in adj5 centre) or (drop in adj5 centre) or (drop-in adj5 centres) or (drop in adj5 centres)).mp. |
| 40. ((drop-in adj5 clinic) or (drop in adj5 clinic) or (drop-in adj5 clinics) or (drop in adj5 clinics)).mp. |
| 41. ((out-of-hours adj5 service) or (out-of-hours adj5 services)).mp. |
| 42. ((out of hours adj5 service) or (out of hours adj5 services)).mp. |
| 43. 5 or 6 or 7 or 8 or 9 or 10 or 11 or 12 or 13 or 14 or 15 or 16 or 17 or 18 or 19 or 20 or 21 or 22 or 23 or 24 or 25 or 26 or 27 or 28 or 29 or 30 or 31 or 32 or 33 or 34 or 35 or 36 or 37 or 38 or 39 or 40 or 41 or 42 |
| 44. 4 or 43 |
| 45. asthma.mp. or exp ASTHMA/ or exp MODERATE PERSISTENT ASTHMA/ or exp SEVERE PERSISTENT ASTHMA/ |
| 46. chronic obstructive lung disease.mp. or exp Chronic Obstructive Lung Disease/ or COPD.mp. or (COAD adj5 airways).mp. |
| 47. (cardiovascular disease or cardiovascular diseases).mp. or exp Cardiovascular Disease/ |
| 48. diabetes mellitus/ or diabetic coma/ or diabetic foot/ or diabetic hypertension/ or diabetic ketoacidosis/ or diabetic macular edema/ or diabetic nephropathy/ or diabetic neuropathy/ or diabetic obesity/ or diabetic retinopathy/ or insulin dependent diabetes mellitus/ or non insulin dependent diabetes mellitus/ or diabetic angiopathy/ |
| 49. (Long-term conditions or long term conditions).mp. |
| 50. (long-term health problems or long term health problems).mp. [mp=title, original title, abstract, name of substance word, subject heading word] |
| 51. (chronic disease or chronic diseases).mp. or exp Chronic Disease/ |
| 52. (chronic illness or chronic illnesses).mp. [mp=title, original title, abstract, name of substance word, subject heading word] |
| 53. 45 or 46 or 47 or 48 or 49 or 50 or 51 or 52 |
| 54. longitudinal study.mp. or exp Longitudinal Study/ |
| 55. prospective study.mp. or exp Prospective Study/ |
| 56. exp Randomized Controlled Trial/ or randomized controlled trial.mp. or randomised controlled trial.mp. |
| 57. 54 or 55 or 56 |
| 58. 44 and 53 and 57 |

**EBM Reviews - Cochrane Database of Systematic Reviews**

Using ADJn operator

| 1. (healthcare adj5 utilization).mp. [mp=title, original title, abstract, name of substance word, subject heading word] |
| --- |
| 2. (healthcare adj5 utilisation).mp. |
| 3. (health care adj5 utilization).mp. [mp=title, original title, abstract, name of substance word, subject heading word] |
| 4. (health care adj5 utilisation).mp. |
| 5. (healthcare adj5 costs).mp. [mp=title, original title, abstract, name of substance word, subject heading word] |
| 6. (health care adj5 costs).mp. [mp=title, original title, abstract, name of substance word, subject heading word] |
| 7. (hospital adj5 utilization).mp. [mp=title, original title, abstract, name of substance word, subject heading word] |
| 8. (hospital adj5 utilisation).mp. |
| 9. (outpatient department adj5 utilization).mp. [mp=title, original title, abstract, name of substance word, subject heading word] |
| 10. (outpatient department adj5 utilisation).mp. |
| 11. (emergency health services adj5 utilization).mp. [mp=title, original title, abstract, name of substance word, subject heading word] |
| 12. (emergency health services adj5 utilisation).mp. |
| 13. (emergency ward adj5 utilization).mp. [mp=title, original title, abstract, name of substance word, subject heading word] |
| 14. (emergency ward adj5 utilisation).mp. |
| 15. (emergency care adj5 utilization).mp. [mp=title, original title, abstract, name of substance word, subject heading word] |
| 16. (emergency care adj5 utilisation).mp. |
| 17. (emergency service adj5 utilization).mp. [mp=title, original title, abstract, name of substance word, subject heading word] |
| 18. (emergency service adj5 utilisation).mp. |
| 19. (emergency medical services adj5 utilization).mp. [mp=title, original title, abstract, name of substance word, subject heading word] |
| 20. (emergency medical services adj5 utilisation).mp. |
| 21. ((accident and emergency) adj5 utilization).mp. [mp=title, original title, abstract, name of substance word, subject heading word] |
| 22. ((accident and emergency) adj5 utilisation).mp. |
| 23. (unscheduled care adj5 utilization).mp. [mp=title, original title, abstract, name of substance word, subject heading word] |
| 24. (unscheduled care adj5 utilisation).mp. |
| 25. (unscheduled care adj5 costs).mp. [mp=title, original title, abstract, name of substance word, subject heading word] |
| 26. (community care adj5 utilization).mp. [mp=title, original title, abstract, name of substance word, subject heading word] |
| 27. (community care adj5 utilisation).mp. |
| 28. (community health services adj5 utilization).mp. [mp=title, original title, abstract, name of substance word, subject heading word] |
| 29. (community health services adj5 utilisation).mp. |
| 30. (primary health care adj5 utilization).mp. [mp=title, original title, abstract, name of substance word, subject heading word] |
| 31. (primary health care adj5 utilisation).mp. |
| 32. (primary healthcare adj5 utilization).mp. |
| 33. (primary healthcare adj5 utilisation).mp. |
| 34. (mental health services adj5 utilization).mp. [mp=title, original title, abstract, name of substance word, subject heading word] |
| 35. (mental health services adj5 utilisation).mp. |
| 36. ((walk-in adj5 centre) or (walk in adj5 centre) or (walk-in adj5 centres) or (walk in adj5 centres)).mp. |
| 37. ((walk-in adj5 clinic) or (walk in adj5 clinic) or (walk-in adj5 clinics) or (walk in adj5 clinics)).mp. |
| 38. ((drop-in adj5 centre) or (drop in adj5 centre) or (drop-in adj5 centres) or (drop in adj5 centres)).mp. |
| 39. ((drop-in adj5 clinic) or (drop in adj5 clinic) or (drop-in adj5 clinics) or (drop in adj5 clinics)).mp. |
| 40. ((out-of-hours adj5 service) or (out-of-hours adj5 services)).mp. |
| 41. ((out of hours adj5 service) or (out of hours adj5 services)).mp. |
| 42. 1 or 3 or 4 or 5 or 6 or 7 or 8 or 9 or 10 or 11 or 12 or 13 or 14 or 15 or 16 or 17 or 18 or 19 or 20 or 21 or 22 or 23 or 24 or 25 or 26 or 27 or 28 or 29 or 30 or 31 or 32 or 33 or 34 or 35 or 36 or 37 or 38 or 39 or 40 or 41 |
| 43. asthma.mp. [mp=title, original title, abstract, name of substance word, subject heading word] |
| 44. chronic obstructive pulmonary disease.mp. [mp=title, original title, abstract, name of substance word, subject heading word] |
| 45. (COPD or (COAD adj5 airways)).mp. [mp=title, original title, abstract, name of substance word, subject heading word] |
| 46. cardiovascular disease.mp. [mp=title, original title, abstract, name of substance word, subject heading word] |
| 47. diabetes.mp. [mp=title, original title, abstract, name of substance word, subject heading word] |
| 48. (Long-term conditions or long term conditions).mp. |
| 49. (Long-term health problems or long term health problems).mp. |
| 50. chronic disease.mp. |
| 51. chronic diseases.mp. |
| 52. (Chronic disease adj5 management).mp. |
| 53. (Chronic illness or chronic illnesses).mp. |
| 54. 43 or 44 or 45 or 46 or 47 or 48 or 49 or 50 or 51 or 52 or 53 |
| 55. 42 and 54 |

**Cochrane Central Register of Controlled Trials search strategy**

Using ADJn operator

| 1. exp Health Care Costs/ |
| --- |
| 2. exp "cost of illness"/ |
| 3. (health care adj5 costs).mp. [mp=title, original title, abstract, name of substance word, subject heading word] |
| 4. (health care adj5 utilization).mp. [mp=title, original title, abstract, name of substance word, subject heading word] |
| 5. (health care adj5 utilisation).mp. [mp=title, original title, abstract, name of substance word, subject heading word] |
| 6. (healthcare adj5 costs).mp. [mp=title, original title, abstract, name of substance word, subject heading word] |
| 7. (healthcare adj5 utilization).mp. [mp=title, original title, abstract, name of substance word, subject heading word] |
| 8. (healthcare adj5 utilisation).mp. [mp=title, original title, abstract, name of substance word, subject heading word] |
| 9. (hospital adj5 utilization).mp. [mp=title, original title, abstract, name of substance word, subject heading word] |
| 10. (hospital adj5 utilisation).mp. [mp=title, original title, abstract, name of substance word, subject heading word] |
| 11. (outpatient department adj5 utilization).mp. [mp=title, original title, abstract, name of substance word, subject heading word] |
| 12. (outpatient department adj5 utilisation).mp. [mp=title, original title, abstract, name of substance word, subject heading word] |
| 13. (emergency health services adj5 utilization).mp. [mp=title, original title, abstract, name of substance word, subject heading word] |
| 14. (emergency health services adj5 utilisation).mp. [mp=title, original title, abstract, name of substance word, subject heading word] |
| 15. (emergency ward adj5 utilization).mp. [mp=title, original title, abstract, name of substance word, subject heading word] |
| 16. (emergency ward adj5 utilisation).mp. [mp=title, original title, abstract, name of substance word, subject heading word] |
| 17. (emergency care adj5 utilization).mp. [mp=title, original title, abstract, name of substance word, subject heading word] |
| 18. (emergency care adj5 utilisation).mp. [mp=title, original title, abstract, name of substance word, subject heading word] |
| 19. (emergency service adj5 utilization).mp. [mp=title, original title, abstract, name of substance word, subject heading word] |
| 20. (emergency service adj5 utilisation).mp. [mp=title, original title, abstract, name of substance word, subject heading word] |
| 21. (emergency medical services adj5 utilization).mp. [mp=title, original title, abstract, name of substance word, subject heading word] |
| 22. (emergency medical services adj5 utilisation).mp. [mp=title, original title, abstract, name of substance word, subject heading word] |
| 23. ((accident and emergency) adj5 utilization).mp. [mp=title, original title, abstract, name of substance word, subject heading word] |
| 24. ((accident and emergency) adj5 utilisation).mp. [mp=title, original title, abstract, name of substance word, subject heading word] |
| 25. (unscheduled care adj5 utilization).mp. [mp=title, original title, abstract, name of substance word, subject heading word] |
| 26. (unscheduled care adj5 utilisation).mp. [mp=title, original title, abstract, name of substance word, subject heading word] |
| 27. (unscheduled care adj5 costs).mp. [mp=title, original title, abstract, name of substance word, subject heading word] |
| 28. (community care adj5 utilization).mp. [mp=title, original title, abstract, name of substance word, subject heading word] |
| 29. (community care adj5 utilisation).mp. [mp=title, original title, abstract, name of substance word, subject heading word] |
| 30. (primary health care adj5 utilization).mp. [mp=title, original title, abstract, name of substance word, subject heading word] |
| 31. (primary healthcare adj5 utilization).mp. |
| 32. (primary health care adj5 utilisation).mp. [mp=title, original title, abstract, name of substance word, subject heading word] |
| 33. (primary healthcare adj5 utilisation).mp. |
| 34. (mental health services adj5 utilization).mp. [mp=title, original title, abstract, name of substance word, subject heading word] |
| 35. (mental health services adj5 utilisation).mp. [mp=title, original title, abstract, name of substance word, subject heading word] |
| 36. ((walk-in adj5 centre) or (walk in adj5 centre) or (walk-in adj5 centres) or (walk in adj5 centres)).mp. |
| 37. ((walk-in adj5 clinic) or (walk in adj5 clinic) or (walk-in adj5 clinics) or (walk in adj5 clinics)).mp. |
| 38. ((drop-in adj5 centre) or (drop in adj5 centre) or (drop-in adj5 centres) or (drop in adj5 centres)).mp. |
| 39. ((drop-in adj5 clinic) or (drop in adj5 clinic) or (drop-in adj5 clinics) or (drop in adj5 clinics)).mp. |
| 40. ((out-of-hours adj5 service) or (out of hours adj5 service)).mp. |
| 41. ((out-of-hours adj5 services) or (out of hours adj5 services)).mp. |
| 42. 1 or 2 or 3 or 4 or 5 or 6 or 7 or 8 or 9 or 10 or 11 or 12 or 13 or 14 or 15 or 16 or 17 or 18 or 19 or 20 or 21 or 22 or 23 or 24 or 25 or 26 or 27 or 28 or 29 or 30 or 31 or 32 or 33 or 34 or 35 or 36 or 37 or 38 or 39 or 40 or 41 |
| 43. exp Asthma/ |
| 44. exp Pulmonary Disease, Chronic Obstructive/ |
| 45. exp Cardiovascular Diseases/ |
| 46. exp Diabetes Mellitus/ |
| 47. asthma.mp. [mp=title, original title, abstract, name of substance word, subject heading word] |
| 48. chronic obstructive pulmonary disease.mp. [mp=title, original title, abstract, name of substance word, subject heading word] |
| 49. (COPD or (COAD adj5 airways)).mp. [mp=title, original title, abstract, name of substance word, subject heading word] |
| 50. cardiovascular disease.mp. [mp=title, original title, abstract, name of substance word, subject heading word] |
| 51. cardiovascular diseases.mp. [mp=title, original title, abstract, name of substance word, subject heading word] |
| 52. diabetes.mp. [mp=title, original title, abstract, name of substance word, subject heading word] |
| 53. (Long-term conditions or long term conditions).mp. |
| 54. (Long-term health problems or long term health problems).mp. |
| 55. chronic disease.mp. |
| 56. chronic diseases.mp. |
| 57. (Chronic disease adj5 management).mp. |
| 58. (Chronic illness or chronic illnesses).mp. |
| 59. 43 or 44 or 45 or 46 or 47 or 48 or 49 or 50 or 51 or 52 or 53 or 54 or 55 or 56 or 57 or 58 |
| 60. 42 and 59 |

**Psychinfo search strategy**

With ADJn operator, withOUT methodological constraints

| 1. exp health care utilization/ |
| --- |
| 2. exp health care costs/ |
| 3. exp HOSPITALIZATION/ |
| 4. 1 or 2 or 3 |
| 5. (health care services adj5 utilization).mp. |
| 6. (health care services adj5 utilisation).mp. |
| 7. (healthcare services adj5 utilization).mp. |
| 8. (healthcare services adj5 utilisation).mp. |
| 9. (healthcare adj5 utilization).mp. [mp=title, abstract, heading word, table of contents, key concepts] |
| 10. (health care adj5 utilization).mp. |
| 11. (healthcare adj5 utilisation).mp. |
| 12. (health care adj5 utilisation).mp. |
| 13. (hospital adj5 utilization).mp. |
| 14. (hospital adj5 utilisation).mp. |
| 15. (outpatients adj5 utilization).mp. [mp=title, abstract, heading word, table of contents, key concepts] |
| 16. (outpatients adj5 utilisation).mp. |
| 17. (emergency services adj5 utilization).mp. [mp=title, abstract, heading word, table of contents, key concepts] |
| 18. (emergency services adj5 utilisation).mp. |
| 19. (emergency care adj5 utilization).mp. [mp=title, abstract, heading word, table of contents, key concepts] |
| 20. (emergency care adj5 utilisation).mp. |
| 21. (unscheduled care adj5 utilization).mp. [mp=title, abstract, heading word, table of contents, key concepts] |
| 22. (unscheduled care adj5 utilisation).mp. |
| 23. (unscheduled care adj5 costs).mp. [mp=title, abstract, heading word, table of contents, key concepts] |
| 24. (community services adj5 utilization).mp. [mp=title, abstract, heading word, table of contents, key concepts] |
| 25. (community services adj5 utilisation).mp. |
| 26. (primary health care adj5 utilization).mp. [mp=title, abstract, heading word, table of contents, key concepts] |
| 27. (primary healthcare adj5 utilization).mp. |
| 28. (primary health care adj5 utilisation).mp. |
| 29. (primary healthcare adj5 utilisation).mp. |
| 30. (mental health services adj5 utilization).mp. [mp=title, abstract, heading word, table of contents, key concepts] |
| 31. (mental health services adj5 utilisation).mp. |
| 32. ((walk-in adj5 centre) or (walk in adj5 centre) or (walk-in adj5 centres) or (walk in adj5 centres)).mp. |
| 33. ((walk-in adj5 clinic) or (walk in adj5 clinic) or (walk-in adj5 clinics) or (walk in adj5 clinics)).mp. |
| 34. ((drop-in adj5 centre) or (drop in adj5 centre) or (drop-in adj5 centres) or (drop in adj5 centres)).mp. |
| 35. ((drop-in adj5 clinic) or (drop in adj5 clinic) or (drop-in adj5 clinics) or (drop in adj5 clinics)).mp. |
| 36. ((out-of-hours adj5 service) or (out-of-hours adj5 services)).mp. |
| 37. ((out of hours adj5 service) or (out of hours adj5 services)).mp. |
| 38. 5 or 6 or 7 or 8 or 9 or 10 or 11 or 12 or 13 or 14 or 15 or 16 or 17 or 18 or 19 or 20 or 21 or 22 or 23 or 24 or 25 or 26 or 27 or 28 or 29 or 30 or 31 or 32 or 33 or 34 or 35 or 36 or 37 |
| 39. 4 or 38 |
| 40. diabetes.mp. or exp DIABETES/ or exp DIABETES MELLITUS/ |
| 41. (lung disorders or chronic obstructive pulmonary disease or COPD).mp. or exp Lung Disorders/ or COPD.mp. or (COAD adj5 airways).mp. |
| 42. (cardiovascular disease or cardiovascular diseases).mp. or exp Cardiovascular Disorders/ |
| 43. asthma.mp. or exp ASTHMA/ |
| 44. (Long-term conditions or long term conditions).mp. |
| 45. (Long-term health problems or long term health problems).mp. |
| 46. exp Chronic Illness/ or chronic disease.mp. or chronic illness.mp. or chronic diseases.mp. or chronic illnesses.mp. |
| 47. (Chronic disease adj5 management).mp. |
| 48. 40 or 41 or 42 or 43 or 44 or 45 or 46 |
| 49. 39 and 48 |

**British Nursing Index search strategy**

Using ADJn operator withOUT methodological constraints.

| 1. (primary health care adj5 utilization).mp. [mp=ti, ab, hw] |
| --- |
| 2. (primary health care adj5 utilisation).mp. |
| 3. (community care adj5 utilization).mp. [mp=ti, ab, hw] |
| 4. (community care adj5 utilisation).mp. |
| 5. (community psychiatric nursing adj5 utilization).mp. [mp=ti, ab, hw] |
| 6. (community psychiatric nursing adj5 utilisation).mp. |
| 7. (health care teams adj5 utilization).mp. [mp=ti, ab, hw] |
| 8. (health care teams adj5 utilisation).mp. |
| 9. (health provision adj5 utilization).mp. [mp=ti, ab, hw] |
| 10. (health provision adj5 utilisation).mp. |
| 11. (mental health community care adj5 utilization).mp. [mp=ti, ab, hw] |
| 12. (mental health community care adj5 utilisation).mp. |
| 13. (community health services adj5 utilization).mp. [mp=ti, ab, hw] |
| 14. (community health services adj5 utilisation).mp. |
| 15. (mental health services health services adj5 utilization).mp. [mp=ti, ab, hw] |
| 16. (mental health services health services adj5 utilisation).mp. |
| 17. exp Patients Admission/ |
| 18. (healthcare adj5 utilization).mp. [mp=ti, ab, hw] |
| 19. (healthcare adj5 utilisation).mp. |
| 20. (health care adj5 utilization).mp. |
| 21. (health care adj5 utilisation).mp. |
| 22. (outpatients department adj5 utilization).mp. [mp=ti, ab, hw] |
| 23. (outpatients department adj5 utilisation).mp. |
| 24. (hospital adj5 utilization).mp. [mp=ti, ab, hw] |
| 25. (hospital adj5 utilisation).mp. |
| 26. (emergency care adj5 utilization).mp. [mp=ti, ab, hw] |
| 27. (emergency care adj5 utilisation).mp. |
| 28. (emergency service adj5 utilization).mp. [mp=ti, ab, hw] |
| 29. (emergency service adj5 utilisation).mp. |
| 30. (emergency services adj5 utilization).mp. [mp=ti, ab, hw] |
| 31. (emergency services adj5 utilisation).mp. |
| 32. (emergency medical services adj5 utilization).mp. [mp=ti, ab, hw] |
| 33. (emergency medical services adj5 utilisation).mp. |
| 34. ((accident and emergency) adj5 utilization).mp. [mp=ti, ab, hw] |
| 35. ((accident and emergency) adj5 utilisation).mp. |
| 36. (unscheduled care adj5 utilization).mp. [mp=ti, ab, hw] |
| 37. (unscheduled care adj5 utilisation).mp. |
| 38. ((walk-in adj5 centre) or (walk in adj5 centre) or (walk-in adj5 centres) or (walk in adj5 centres)).mp. |
| 39. ((walk-in adj5 clinic) or (walk in adj5 clinic) or (walk-in adj5 clinics) or (walk in adj5 clinics)).mp. |
| 40. ((drop-in adj5 centre) or (drop in adj5 centre) or (drop-in adj5 centres) or (drop in adj5 centres)).mp. |
| 41. ((drop-in adj5 clinic) or (drop in adj5 clinic) or (drop-in adj5 clinics) or (drop in adj5 clinics)).mp. |
| 42. ((out-of-hours adj5 service) or (out-of-hours adj5 services)).mp. |
| 43. ((out of hours adj5 service) or (out of hours adj5 services)).mp. |
| 44. 1 or 2 or 3 or 4 or 5 or 6 or 7 or 8 or 9 or 10 or 11 or 12 or 13 or 14 or 15 or 16 or 17 or 18 or 19 or 20 or 21 or 22 or 23 or 24 or 25 or 26 or 27 or 28 or 29 or 30 or 31 or 32 or 33 or 34 or 35 or 36 or 37 or 38 or 39 or 40 or 41 or 42 or 43 |
| 45. asthma.mp. or exp ASTHMA/ |
| 46. (chronic obstructive pulmonary disease or COPD).mp. or exp COPD/ or (COAD adj5 airways).mp. |
| 47. exp "cardiovascular system and disorders"/ or cardiovascular disease.mp. or cardiovascular diseases.mp. |
| 48. diabetes.mp. or exp DIABETES/ |
| 49. diabetes complications/ |
| 50. (Long-term conditions or long term conditions).mp. |
| 51. (Long-term health problems or long term health problems).mp. |
| 52. Chronic Disease/ or chronic disease.mp. or chronic diseases.mp. or chronic illness.mp. or chronic illnesses.mp. |
| 53. (Chronic disease adj5 management).mp. |
| 54. 45 or 46 or 47 or 48 or 49 or 50 or 51 or 52 or 53 |
| 55. 44 and 54 |

# 3.Additional information provided by authors

| **Study Number** | **Author contacted** | **Information provided** | **Outcome for paper** |
| --- | --- | --- | --- |
| 78 | Sneider | Confirmed hospitalizations were urgent | Included |
| 133 | Gudmunsson | Confirmed hospitalizations were urgent | Included |
| 144 | Greaves | Confirmed hospitalizations were urgent | Included |
| 182 | Grace | Confirmed hospitalizations were urgent | Included |
| 215 | Kaptein | Confirmed hospitalizations were urgent | Included |
| 2487 | Gelsomino | Unable to confirm hospitalizations were urgent | Excluded |
| 1744 | Rasekaba | Unable to provide data for anxiety | Excluded |
| 1427 | Schembri | Unable to confirm hospitalizations urgent and provide separate anxiety data | Excluded |
| 1363 | Prina | Unable to provide separate anxiety results | Excluded |
| 1274 | Moser | Unable to provide separate readmission rate results | Excluded |
| 2959 | Mourad | Unable to provide data for anxiety | Excluded |
| 1276 | Mudge | Unable to provide separate anxiety results | Excluded |
| 1158 | Laurin | Unable to provide separate anxiety results | Excluded |
| 502 | Boulanger | Unable to provide separate anxiety results | Excluded |
|  | Coventry | Provided data for unscheduled care use of patients diagnosed with anxiety, and patients not diagnosed with anxiety | Included |

# 4 Forrest Plot Anxiety and unscheduled care, without Abrams

#
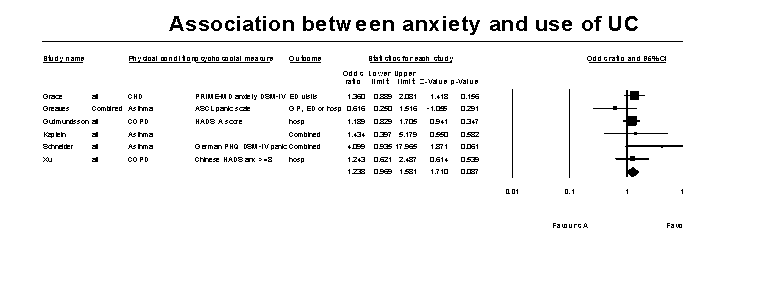


# 5 Funnel Plot of Precision by Log odds ration

#

# 6 Duvall & Tweedie Trim & Fill Procedure Funnel Plot


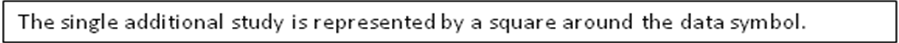


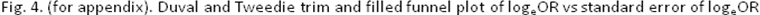


# 7 Formulae used in meta-analysis

[Taken from Comprehensive meta-analysis (version 2.2.048, Nov 7^th^ 2008)]

**1) Calculation of Odds Ratios**

Starting with data in a 2x2 table such that:

A = Number of anxious subjects using unscheduled care

B = Total number of anxious subjects - number of anxious subjects using unscheduled care

C = Number of non-anxious subjects using unscheduled care

D = Total number of non-anxious - number of non-anxious subjects using unscheduled care

Odds ratio = (A x D) / (B x C)

Log odds ratio = log [(A x D) / (B x C)]

Log Odds variance = (1/A + 1/B + 1/C + 1/D)

Log Odds Standard Error = Sq. root (Log Odds Variance)

**2) When results are presented in included paper as continuous variables, the odds ratio was calculated from the standardised mean difference (d) using the following formulae.**

d = (mean in group 1 – mean in group 2)/ pooled standard deviation

Log Odds Ratio = Pi x d / (Sq. root 3), where Pi = 3.14159265358979

Odds Ratio = Exp (Log Odds Ratio)

Log Odds Standard Error = Sq. root (Pi^2^ x d^2^/3)

**3) When results are presented as p-values with group sizes**

Starting with Sample size (N1 and N2) and p-value

Standardized difference in means

p = p-entered / tails

df = N1 + N2 - 2

t= Abs (t for p, df)

Harmonic N = (2 x N1 x N2) / (N1 + N2)

d = t / [Square root (Harmonic N) / Square root (2)]

Standard error of d = Square root [1 / N1 + 1 / N2 + d^2^ / (2 x (N1 + N2))]

Then as 2) above to calculate OR

n.b. where paper fails to state whether p was 1 or 2-tailed, a conservative assumption, that it was 1-tailed, was made if p <0.5.

**4) When results are presented as the correlation between depression and urgent healthcare use.**

Starting with Correlation or Fisher's Z

First compute the correlation ratio, and then convert to standardised mean difference (d).

d = 2 x Correlation coefficient / Square root [1 – (Correlation coefficient^2^)]

Standard error of d = Square root [4 x Correlation SE^2^ / ((1 - Correlation^2^)^3^)]

Then as 2) above to calculate OR

**Pooling of effects from individual studies**

Odds ratios were pooled using the DerSimonian and Laird random effects method {2011 1263 /id}, where the variance of effect sizes is calculated using the following formula.

Variance (r^2^) = Q- (k-1)/Σw_i_ - (Σw_i_^2^/Σw_i_)

Where Q = the heterogeneity statistic = Σw_i_ (θ_i_ - θ_IV_)^2^

k is the number of studies

w_i_ is the inverse variance weight for study i = (1/SE^2^)

θ_i_ is the effect size for study i,

θ_IV_ = the inverse variance weighted pooled effect size (Σw_i_θ_i_ / Σw_i_)

The weight for each study (*w_i_) is then calculated = 1/ [SE (θ_i_)^2^ + r^2^]

The DerSimonian Laird (θ_DL_) effect size is then calculated as follows:

(θ_DL_) = (Σ*w_i_θ_i_ / Σ*w_i_)
